# Non-Digestible Oligosaccharides Can Suppress Basophil Degranulation in Whole Blood of Peanut-Allergic Patients

**DOI:** 10.3389/fimmu.2018.01265

**Published:** 2018-06-11

**Authors:** Simone M. Hayen, Constance F. den Hartog Jager, André C. Knulst, Edward F. Knol, Johan Garssen, Linette E. M. Willemsen, Henny G. Otten

**Affiliations:** ^1^Department of Dermatology/Allergology, University Medical Center Utrecht, Utrecht University, Utrecht, Netherlands; ^2^Laboratory of Translational Immunology, University Medical Center Utrecht, Utrecht, Netherlands; ^3^Division of Pharmacology, Faculty of Science, Utrecht Institute for Pharmaceutical Sciences, Utrecht University, Utrecht, Netherlands; ^4^Nutricia Research, Immunology, Utrecht, Netherlands

**Keywords:** basophil degranulation, food allergy, galectin-9, immunomodulation, non-digestible oligosaccharides

## Abstract

**Background:**

Dietary non-digestible oligosaccharides (NDOs) have a protective effect against allergic manifestations in children at risk. Dietary intervention with NDOs promotes the colonization of beneficial bacteria in the gut and enhances serum galectin-9 levels in mice and atopic children. Next to this, NDOs also directly affect immune cells and low amounts may reach the blood. We investigated whether pre-incubation of whole blood from peanut-allergic patients with NDOs or galectin-9 can affect basophil degranulation.

**Methods:**

Heparinized blood samples from 15 peanut-allergic adult patients were pre-incubated with a mixture of short-chain galacto-oligosaccharides and long-chain fructo-oligosaccharides (scGOS/lcFOS), scFOS/lcFOS, or galectin-9 (1 or 5 µg/mL) at 37°C in the presence of IL-3 (0.75 ng/mL). After 2, 6, or 24 h, a basophil activation test was performed. Expression of FcεRI on basophils, plasma cytokine, and chemokine concentrations before degranulation were determined after 24 h.

**Results:**

Pre-incubation with scGOS/lcFOS, scFOS/lcFOS, or galectin-9 reduced anti-IgE-mediated basophil degranulation. scFOS/lcFOS or 5 µg/mL galectin-9 also decreased peanut-specific basophil degranulation by approximately 20%, mainly in whole blood from female patients. Inhibitory effects were not related to diminished FcεRI expression on basophils. Galectin-9 was increased in plasma after pre-incubation with scGOS/lcFOS, and both NDOs and 5 µg/mL galectin-9 increased MCP-1 production.

**Conclusion and clinical relevance:**

The prebiotic mixture scFOS/lcFOS and galectin-9 can contribute to decreased degranulation of basophils *in vitro* in peanut-allergic patients. The exact mechanism needs to be elucidated, but these NDOs might be useful in reducing allergic symptoms.

## Introduction

In Westernized countries, the prevalence of food allergies has increased over the years and is still increasing (1, 2). The prevalence of food allergy is currently estimated between 6 and 10% (3). Among children, less is known about the prevalence, although peanut allergy is one of the most common food allergies. Allergic reactions develop as a result of a hampered tolerance mechanism toward harmless antigens (4). When patients are sensitized, B cells start to produce antigen-specific IgE molecules that can sensitize the high-affinity FcεRI on mast cell or basophils (5). Upon a second encounter with the specific allergen, these IgE molecules can crosslink and will induce degranulation of mast cells and basophils, leading to clinical symptoms due to the release of histamine and other mediators. Currently, there is no curative treatment available to re-establish tolerance against these harmless food antigens, although progress is made in terms of immunotherapy and dietary adjunct therapy with, for example, probiotics that can improve the efficacy of immunotherapy (6).

Previous research has indicated a role of prebiotic non-digestible oligosaccharides (NDOs) in decreasing the incidence of atopic dermatitis in children at risk of developing allergy (7–9). The exact mechanism of action of these NDOs is not fully understood, however, it is known that they can promote the colonization of beneficial bacteria in the gut, similar as human milk oligosaccharides (HMOs) in breast milk (10, 11). Whey-allergic mice receiving oral immunotherapy in combination with a diet of short- and long-chain fructo-oligosaccharides (scFOS/lcFOS) experienced enhanced serum galectin-9 levels (12). Galectin-9 is a soluble type lectin which can, among others, be released by intestinal epithelial cells. It can bind to carbohydrate moieties located on the heavy chains of IgE (13, 14), hereby suppressing degranulation of mast cells and basophils by the inhibition of the formation of the IgE-allergen complex, which could be abrogated in the presence of lactose (14, 15). In addition, galectin-9 can support tolerance *via* the induction of Tregs (15, 16). Next to induction of galectin-9, these NDOs might also have a direct effect on immune cells. Earlier research indicated that HMOs (normally present in concentrations of 5–23 g/L in human milk) could be traced with ^13^C labeling, HPLC, and other techniques in plasma and urine (17–20). Approximately 0.05–0.1% of these oligosaccharides could be traced back to plasma, while 4% was traced back in urine (18–21). In addition, a study with fructo-oligosaccharides demonstrated that FOS, and hereby most likely more prebiotic structures, could reach the plasma compartment and were excreted in the urine (21).

For this research, we were interested in both the direct and indirect (galectin-9) effects of NDOs on basophil degranulation in whole blood of peanut-allergic patients. Therefore, two different mixtures of NDOs were tested; short-chain galacto-oligosaccharides and long-chain fructo-oligosaccharides (scGOS/lcFOS) and scFOS/lcFOS. In addition, the effects of galectin-9 on basophil degranulation were assessed. Next to determining the effects on basophil degranulation, the expression of FcεRI on basophils and mediator release in whole blood exposed to NDOs or galectin-9 was determined.

## Materials and Methods

### Study Design and Study Population

Fifteen peanut-allergic patients (6 male and 9 female; age 18–50; mean 32) were recruited from the Department of Dermatology/Allergology at the University Medical Center Utrecht. Inclusion criteria consisted of a type I allergic reaction to peanut, previously confirmed by a positive double-blind placebo-controlled food challenge and serum peanut-specific IgE (Table 1). Exclusion criteria were pregnancy or the continuous use of systemic immune-suppressants, such as prednisone. Total IgE levels were measured by ELISA (Euroimmun, Lübeck). In addition, average basophil percentages are shown per patient (normal range 0–2%). All patients gave written informed consent before enrollment in the study. The study was reviewed and approved by the Ethics Committee of the University Medical Center Utrecht (NL51606.041.15).

**Table 1:** Patient characteristics.

Patient	Age (years)	Sex (M/F)	Müller score^a^	SPT peanut	Subjective effective dose (ED) (mg)	Objective ED (mg)	Total IgE (IU/mL)	CAP peanut (kU/L)	Percentage basophils (% total)
N01	41	F	2	3+	10	–	238	1.7	0.35
N02	37	M	4	3+	0.1	300	1,482	44	0.63
N03	45	M	2	4+	100	–	101	1.8	0.40
N04	50	F	3	4+	10	10	1,537	12	0.38
N05	35	F	4	4+	0.1	–	535	85	0.62
N06	27	F	2	4+	4	40	3,786	12.8	0.26
N07	42	M	3	5+	Not known	300	599	42.7	0.30
N08	24	M	1	4+	100	>3,000	2,054	1.9	0.20
N09	24	F	3	3+	Not known	>3,000	1,062	1	0.34
N10	18	F	3	4+	300	1,000	5,823	>100	0.40
N11	32	F	2	4+	10	3,000	3,941	No data	0.40
N12	27	M	3	5+	0.1	1,000	59,272	66	0.41
N13	25	M	1	3+	10	–	2,302	11.2	0.53
N14	26	F	2	4+	0.1	100	3,287	9.7	0.53
N15	34	F	2	4+	40	12,000	1,347	1.55	0.25

*Age, sex, Müller score, SPT, subjective and objective ED as established by double-blind placebo-controlled food challenge (DBPCFC), total and peanut-specific IgE, and average basophil count per subject*.

*^a^Müller score 0: symptoms oral cavity, 1: symptoms of the skins and mucous membranes, 2: gastro-intestinal symptoms, 3: respiratory symptoms, 4: cardiovascular symptoms*.

*Skin prick test (mm), diameter of 3 mm (3+) is considered positive. All patients underwent a DBPCFC, subjective and objective EDs are displayed in mg*.

### Basophil Activation Test (BAT)

Whole heparinized blood was obtained from 15 peanut-allergic patients. Blood was incubated for 2, 6, or 24 h at 37°C with galectin-9 [1 µg/mL (~28 nM) or 5 µg/mL (~140 nM), R&D Systems, Minneapolis, MN, USA], 0.05% (w/v) of a 9:1 mixture of scGOS (Vivinal GOS, Borculo Domo, the Netherlands) and lcFOS (scGOS/lcFOS) (Raftiline HP, Orafti) or 0.05% of a 9:1 mixture of scFOS (Raftilose P95, Orafti) and lcFOS (scFOS/lcFOS). To maintain basophil viability, 0.75 ng/mL IL-3 (R&D Systems) was added to the blood during pre-incubation and control samples were included. After different pre-incubation periods, a BAT was performed. Basophils in the different blood samples were stimulated for 30 min with increasing concentrations of anti-IgE (0.1, 0.3, and 1 µg/mL, Vector Laboratories, Burlingame, CA, USA) or crude peanut extract (0.1, 0.3, 1, 3, 10, 100, and 1,000 ng/mL) in RPMI 1640 medium (Gibco, Life Technologies) supplemented with 1 ng/mL IL-3. Control samples included RMPI + IL-3 and formyl-methionyl-leucyl-phenylalanine (1 µM fMLP, Sigma-Aldrich). Leukocytes were stained with an antibody cocktail of CD45-PO (Life Technologies), CD123-FITC (BioLegend), HLA-DR-PB (BioLegend), CD63-PE (Monosan), CD41-PE-Cy7 (Beckman Coulter), and CD203c (BioLegend). Basophils were defined as CD45^+^ CD203c^+^ CD123^+^ and HLA-DR^−^ CD41^−^, and degranulation was determined as CD63^+^ cells. Results are expressed as percentage of CD63^+^ cells. Per patient, the dose of peanut allergen or anti-IgE that induced maximal degranulation in the control sample was used to normalize the data.

### Determination of FcεRI on the Cell Surface of Basophils

FcεRI expression was quantified in seven random patients using a QIFIKIT (Dako) according to the manufacturer’s protocol. In short, a small sample of blood was first incubated with a primary mouse monoclonal against FcεRI (clone CRA-1), or an isotype. CRA-1 binds to both the open and occupied FcεRI receptor (22), Next, the blood samples, set-up and calibration beads were labeled with an FITC-conjugated anti-mouse secondary antibody, followed by further staining of the samples with an antibody cocktail to identify basophils (CD45, CD123, HLA-DR, CD203c, and CD41). FcεRI expression was quantified based on the calibration curve from the calibration beads by Graphpad Prism 7.0. Values were calculated relative to the control sample.

### Determination of Cytokines and Chemokines in Plasma Pre-Incubated Samples

Residual plasma was collected after pre-incubation of the blood samples. Mediators secreted by basophils or involved in basophil activation or degranulation were measured (IL-4 (23), IL-5 (24), GM-CSF (25), MCP-1 (26), MDC (27), and TARC [upregulated by basophil derived IL-4 and IL-13 (28)]). Chemoattractants or mediators in basophil degranulation Eotaxin-3 (29), RANTES (30), galectin-3 (31, 32), and galectin-9 (14) were measured in these plasma samples with a luminex assay, performed by the luminex facility located in the UMC Medical Center, Utrecht. Values were calculated relative to the control sample.

### Statistical Analysis

Data are expressed as mean ± SEM. Statistical significance was analyzed with GraphPad Prism 7.0 software (GraphPad Software, San Diego, CA, USA). Normally distributed data were analyzed with a one-way repeated measures ANOVA followed by Bonferroni *post hoc* analysis. Data were considered significant when *P* < 0.05. Demographic data were analyzed with the non-parametric Mann–Whitney test. Correlation was determined with Pearson’s correlation coefficients.

## Results

### Time-Dependent Kinetics Basophil Degranulation Influenced by NDOs and Galectin-9

**Figure 1:**
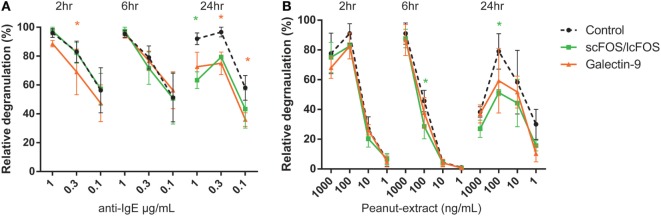
Time-dependent kinetics of non-digestible oligosaccharides or galectin-9 on anti-IgE or CPE induced basophil degranulation of a representative donor. Blood of peanut-allergic patients (*n* = 3) was pre-incubated for three different time-periods with short- and long-chain fructo-oligosaccharides (scFOS/lcFOS) or 1 µg/mL galectin-9 either in the aspecific **(A)** or peanut-specific **(B)** basophil activation test. Data represent *n* = 3 peanut-allergic patients. Significance is indicated compared to the control sample. *P < 0.05 by two-way ANOVA. Green: control compared to scFOS/lcFOS, orange: control compared to galectin-9.

In a pilot experiment, kinetics of the effects of basophil degranulation pre-incubated by NDOs and galectin-9 were determined. A time curve was performed in 1 mL whole blood of three patients. Whole blood samples were pre-incubated for 2, 6, or 24 h at 37°C, with 0.05% scFOS/lcFOS, 1 µg/mL galectin-9 or were left untreated as control. Figure 1A shows the relative IgE-mediated degranulation of basophils of three patients. After 2 and/or 6 h, some decrease in IgE-mediated basophil degranulation and peanut-specific (Figure 1B) was observed in the pre-incubated samples. After 24 h, the differences between the pre-incubated and untreated control sample were most pronounced, therefore we continued with the 24 h pre-incubation.

### Pre-Incubation of Blood With NDOs or Galectin-9 Decreases Basophil Degranulation in Peanut-Allergic Patients

**Figure 2:**
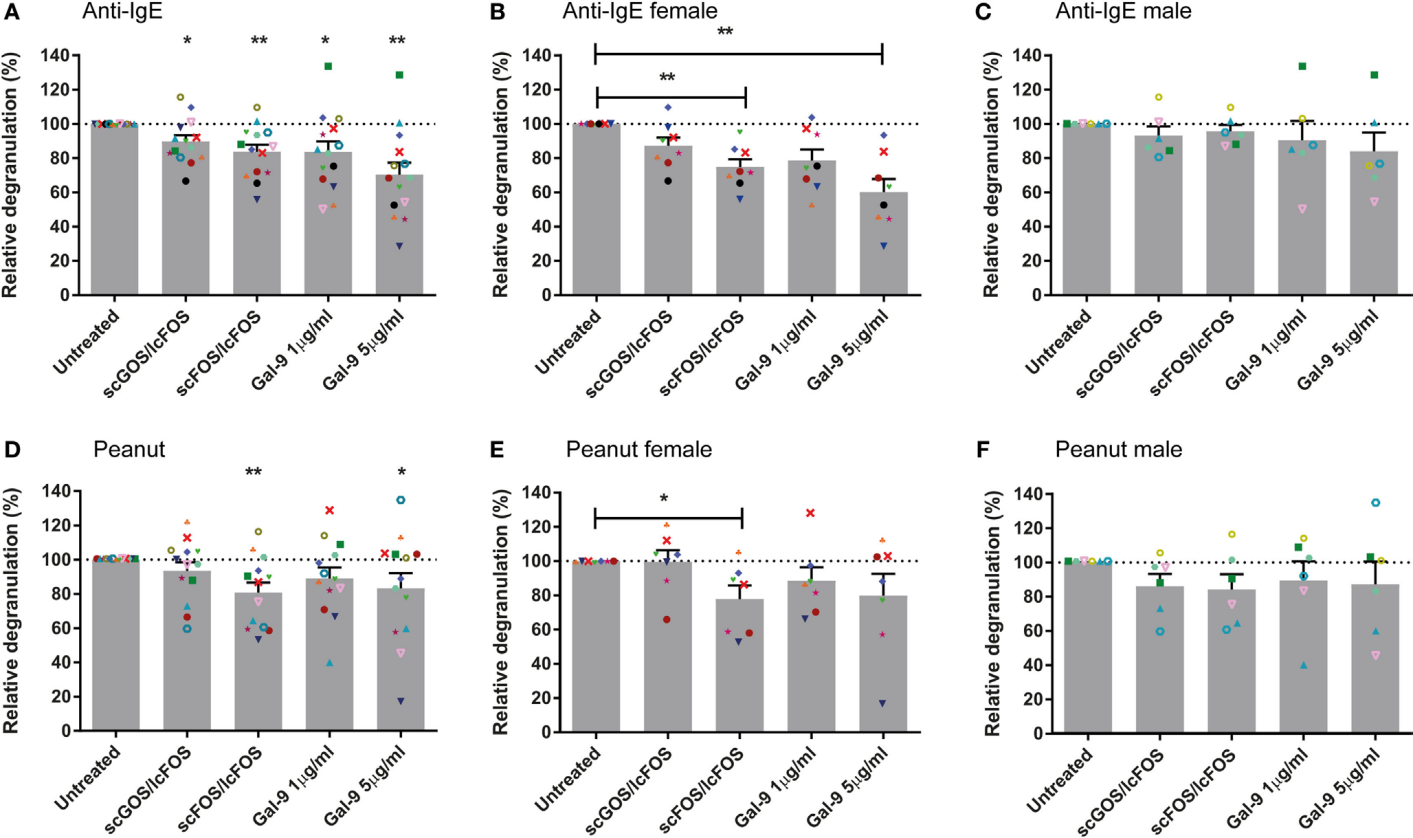
Reduced basophil degranulation after pre-incubation with non-digestible oligosaccharides (NDOs) or galectin-9. Pre-incubation of whole blood for 24 h with NDOs of galectin-9 resulted in a decrease in IgE-mediated basophil degranulation **(A–C)**. Pre-incubation with short- and long-chain fructo-oligosaccharides (scFOS/lcFOS) and 5 µg/mL galectin-9 reduced peanut-specific basophil degranulation **(D–F)**. Females tended to have a higher decrease in basophil degranulation than males after pre-incubation **(B,C,E,F)**. Data represent the mean ± SEM of *n* = 15 peanut-allergic patients, two patients were unresponsive (<5% basophil degranulation) in the peanut-specific basophil activation test (BAT), one of these two patients was also non-responsive in the IgE-mediated BAT. *P < 0.05, **P < 0.01 by one-way ANOVA.

Blood samples pre-incubated with NDOs or galectin-9 showed a reduced basophil degranulation after the IgE-mediated BAT compared to the controls after 24 h (Figure 2A). Pre-incubation with scGOS/lcFOS or scFOS/lcFOS resulted in an average decrease in anti-IgE-mediated basophil degranulation of 11 ± 3.5 or 13 ± 4%, respectively. Pre-incubation with 1 µg/mL galectin-9 resulted in an average decrease of 16 ± 6%, while the highest concentration of galectin-9 could reduce basophil degranulation on average with 30 ± 7%. In the peanut-specific BAT (Figure 2D) only scFOS/lcFOS and the highest dose of galectin-9 reduced basophil degranulation significantly compared to the control sample, with an average reduction of 20 ± 6% (scFOS/lcFOS) and 17 ± 9% (galectin-9). Not all patients were responsive to all pre-incubations, and also differences in response between the aspecific (anti-IgE) and peanut-specific BAT were observed in blood samples of individual patients. In addition, the oligosaccharides did not alter the fMLP-mediated degranulation, indicating an IgE-specific inhibition rather than a general inhibition (data not shown).

### Pre-Incubation of Blood With NDOs or Galectin-9 Reduces Basophil Degranulation More Effective in Female Patients and Does Not Correlate With FcεRI Expression

Next to the role of pre-incubation or the specificity of the BAT (anti-IgE or peanut) on basophil degranulation, differences in degranulation were observed between male and female blood samples. Female blood samples pre-incubated with NDOs or galectin-9 showed less basophil degranulation compared to their control samples than males. This was observed in both the peanut-specific and the anti-IgE BAT (Figures 2B,C,E,F). In the IgE-mediated BAT, significant differences were observed between the female and male samples for scFOS/lcFOS (*P* < 0.01) and a similar trend for galectin-9, 5 µg/mL (*P* < 0.1). In addition, other demographic variables as indicated in Table 2 could not explain the differences between males and females, as they were not statistically different.

**Table 2:** Demographic data of male and female patients.

Characteristic	Male (*n* = 6)	Female (*n* = 7)	*P* value
**Age (years)**			
Mean ± SD	33.3 ± 9.2	31.4 ± 11	0.70
Median (25th, 75th percentile)	32 (25, 43)	27 (24, 41)	
**Müller score**			
Mean ± SD	2.3 ± 1.2	2.4 ± 0.5	0.88
Median (25th, 75th percentile)	2.5 (1, 3.3)	2 (2, 3)	
**CAP peanut (kU/L) ± SD**			
Mean ± SD	27.9 ± 26.7	19.8 ± 35.7	0.36
Median (25th, 75th percentile)	26.9 (1.88, 49.5)	9.7 (1.5, 12.8)	

**Figure 3:**
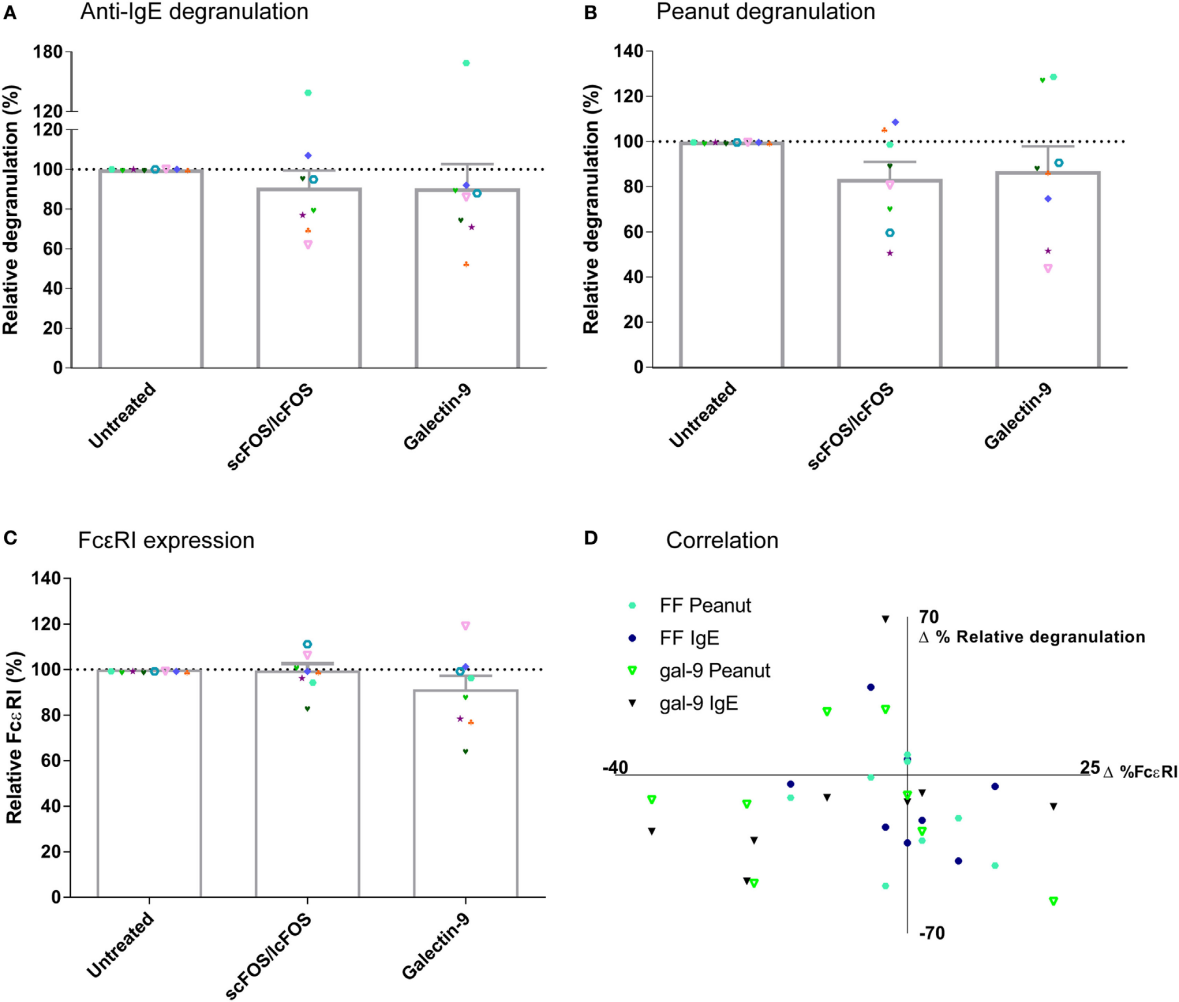
Correlation relative expression of FcεRI and basophil degranulation. Anti-IgE mediated and peanut-specific basophil degranulation after 24 h of pre-incubation with short- and long-chain fructo-oligosaccharides (scFOS/lcFOS) or galectin-9 **(A,B)**. FcεRI expression on basophils was not decreased after pre-incubation with scFOS/lcFOS or 1 µg/mL galectin-9 **(C)**. Δ% relative degranulation was calculated based on panels **(A,B)**, whereas Δ%FcεRI was calculated based on panel **(C)**, both relative to the control samples. No correlation was found between expression of ΔFcεRI on the cell surface and the corresponding Δbasophil degranulation **(D)**. (FF = scFOS/lcFOS). Data represent *n* = 8 patients. Correlation was tested with Pearson correlation coefficient.

To determine whether scFOS/lcFOS or galectin-9 suppressed basophil degranulation *via* directly or indirectly affecting FcεRI expression, these expression levels on basophils were determined in blood samples of seven patients relative to the expression of this receptor on untreated positive control samples of the same donor (Figure 3). First, anti-IgE (Figure 3A) and peanut-specific basophil degranulation (Figure 3B) of blood pre-incubated with either scFOS/lcFOS or galectin-9 was determined as described earlier. In addition, quantitative expression of FcεRI on basophils was determined before basophil degranulation (Figure 3C). No correlation was observed between the difference in expression of FcεRI and the corresponding difference in basophil degranulation of the pre-incubated samples (Figure 3D). Therefore, the mechanism of action of NDOs and galectin-9 cannot be ascribed to effects on expression of FcεRI on the basophil cell surface.

### Luminex Analysis Plasma Samples

**Figure 4:**
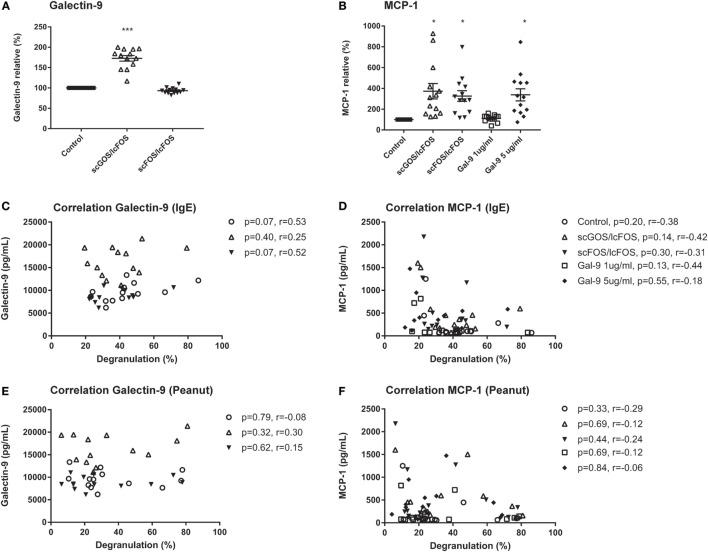
No correlation between relative basophil degranulation and galectin-9 or MCP-1 production. Galectin-9 increased in short-chain galacto-oligosaccharides and long-chain fructo-oligosaccharides (scGOS/lcFOS) pre-incubated samples, and were above the detection limit for galectin-9 treated samples **(A)**. MCP-1 (CCL2) was increased after pre-incubation with both non-digestible oligosaccharides and 5 µg/mL galectin-9 **(B)**. This was not correlated with anti-IgE or peanut-specific basophil degranulation **(C–F)**. Data are represented as mean ± SEM of *n* = 15 patients. *P < 0.05, ***P < 0.001 by one-way ANOVA. Correlation was tested with Pearson correlation coefficient.

To determine whether NDO or galectin-9 pre-incubation of blood affects mediator secretion by cells, remaining plasma samples after the pre-incubation were collected and were analyzed on cytokine and chemokines levels. Table 3 indicates the mean baseline levels per mediator and the mean levels ± SEM when blood samples were pre-incubated with scGOS/lcFOS, scFOS/lcFOS, or galectin-9. IL-5 and GM-CSF were for several samples extrapolated from the standard curve or below the detection limit of the luminex assay, therefore no conclusions can be drawn on the contribution of these mediators in the observed decrease in basophil degranulation. Significant differences compared to the untreated control were found for galectin-9 and MCP-1 (CCL2) levels (Figure 4). Galectin-9 levels in blood pre-incubated with scGOS/lcFOS increased relative to the untreated control sample which was set to 100%, while pre-incubation with scFOS/lcFOS did not result in an increase (Figure 4A). Galectin-9 levels in the galectin-9 pre-incubated samples were above the detection limit of the luminex and are, therefore, not displayed. MCP-1 (CCL2) levels were increased in plasma samples after pre-incubation with scGOS/lcFOS, scFOS/lcFOS, and the highest dose of galectin-9 relative to untreated controls (Figure 4B). However, no correlations were found between levels of galectin-9, MCP-1, and anti-IgE or peanut-specific basophil degranulation (Figures 4C–F). Only a trend between galectin-9 and IgE-mediated basophil degranulation was observed for the control sample and after pre-incubation with scFOS/lcFOS (Figure 4C).

**Table 3:** Average concentration mediators (pg/mL) in blood plasma after 24 h of pre-incubation with non-digestible oligosaccharides.

Mediator	Control ± SEM	scGOS/lcFOS ± SEM	scFOS/lcFOS ± SEM	Galectin-9 (1 µg/mL) ± SEM	Galectin-9 (5 µg/mL) ± SEM
IL-4	4 ± 0.8	3 ± 0.9	3 ± 0.7	3 ± 0.4	4 ± 0.6
IL-5^a^	13 ± 6	11 ± 5	9 ± 3	9 ± 2	11 ± 3
GM-CSF^a^	28 ± 5	23 ± 4	23 ± 3	20 ± 2	25 ± 3
MDC	444 ± 39	458 ± 36	454 ± 33	472 ± 44	507 ± 42
TARC	15 ± 2	15 ± 2	15 ± 2	15 ± 2	15 ± 2
Eotaxin-3	254 ± 48	245 ± 50	251 ± 51	256 ± 53	262 ± 52
RANTES	39,679 ± 4,131	40,454 ± 5,155	39,055 ± 4,537	37,172 ± 4,396	36,854 ± 4,178
Galectin-3	42,093 ± 3,210	35,832 ± 2,878	46,998 ± 9,034	45,240 ± 6,155	37,298 ± 2,584

*^a^Values out of range below*.

*scFOS/lcFOS, short- and long-chain fructo-oligosaccharides; scGOS, short-chain galacto-oligosaccharides*.

## Discussion

Basophil degranulation is an important event in allergic reactions. This study demonstrates that IgE-mediated and peanut-specific basophil degranulation can be reduced by pre-incubation of blood with NDOs scGOS/lcFOS and scFOS/lcFOS, but also by their indirect product galectin-9, which can be enhanced amongst others under influence of NDOs (33). These effects on basophil degranulation were mainly observed in female peanut-allergic subjects. The reduction in basophil degranulation was not correlated with altered FcεRI expression levels on the basophil cell surface. The differences between males and females could not be related to other demographic variables. An explanation for differences between males and females might be hormone-related. While the exact working mechanism of hormones is not elucidated, estrogen receptors for instance are found on several immune subsets and are described to have effects on the allergic sensitization pathway including promotion of basophil degranulation (34–37). More differences in innate and adaptive immune responses between males and females have been studied and described, indicating that sex is an important parameter to consider for the observed differences in basophil degranulation (38). No other relations between the effects of NDOs and other demographic variables, for example age, were observed. However, we cannot exclude this, due to the limited number of patients and their inhomogeneous age distribution.

When blood was pre-incubated with scGOS/lcFOS, an increase in galectin-9 was observed. Soluble type lectin galectin-9 is a small (36 kDa) glycoprotein that can bind to glycans containing galactose and derivatives (39). The carbohydrate recognition domains (CRDs) can only fit galactose residues; other sugar residues do not fit into these CRDs due to steric hindrance (40). Interactions between galactose residues and galectin-9 can result in the formation of so-called lattices which play an important role in the regulation of immune responses (41). We hypothesize that pre-incubation with scGOS/lcFOS stimulates cells in blood, such as T-cells, B-cells, eosinophils, or basophils (14) to release galectin-9. This may result in the reduced basophil degranulation that was observed, since galectin-9 may have bound to IgE on the basophil cell surface, hereby hindering the formation of an IgE-antigen complex. Similar effects of IgE-binding capacities of (recombinant) galectin-9 have previously been described, and here galectin-9 was able to reduce mast cell degranulation in RBL-2H3 cells or HMC-1 cells, which was also explained by steric hindrance by galectin-9 (14, 42). In the allergen-specific BAT, basophil degranulation was not significantly decreased upon pre-incubation with scGOS/lcFOS and the lowest dose of galectin-9. This might be explained by the difference in size between anti-IgE (~150 kDa) and peanut allergens (~15–60 kDa). Peanut-allergens are smaller and have multiple IgE-binding epitopes (43), and therefore may circumvent the steric hindrance caused by galectin-9. The higher concentration of galectin-9 might overcome this steric hindrance in the peanut-specific BAT, while scFOS/lcFOS probably acts *via* a different mechanism than scGOS/lcFOS and galectins.

Although the concentration of galectin-9 was upregulated by scGOS/lcFOS to a concentration of 16 ng/mL, it is relatively low when compared to the recombinant galectin-9 used in this study (1 and 5 µg/mL). No data are available whether such low concentrations of galectin-9 could influence basophil degranulation. Studies that have been performed used higher concentrations, and indicated a dose-response curve for galectin-9 and subsequent degranulation (14, 42). However, these concentrations are always much higher than physiological galectin-9 concentrations in serum of healthy controls (5–12 ng/mL) (44). In addition, most studies performed on basophil degranulation are using recombinant galectin-9, which may also generate different responses than natural galectin-9 that was induced by scGOS/lcFOS. In summary, galectin-9 might be a contributing factor in the observed decrease in degranulation after pre-treatment with scGOS/lcFOS, but since there was no correlation between galectin-9 and degranulation and not all patients were responsive to scGOS/lcFOS, other factors are probably involved.

scFOS/lcFOS was most effective in reducing basophil degranulation in both the IgE-mediated and the peanut-specific BAT, with a similar reduction as 5 µg/mL galectin-9 (approximately 20%). In contrast to scGOS/lcFOS, scFOS/lcFOS did not affect plasma galectin-9 concentrations. This indicates that scFOS/lcFOS may exert its functions in a different manner than scGOS/lcFOS. One of the possible mechanisms by which scFOS/lcFOS may exert its effect is *via* modulation of FcεRI expression. However, no correlation was observed between scFOS/lcFOS induced effects on basophil degranulation and relative FcεRI expression on these cells.

In addition to differences in galectin-9 levels, changes in MCP-1 chemokine levels after pre-incubation were observed. MCP-1 is described in literature as a potent activator for basophil degranulation at concentrations of 3–10 nM and can be produced by various cell types (26, 45). This increase in MCP-1 is in contrast to our findings, since basophil degranulation was reduced in the pre-incubated blood samples. Both NDO mixtures and the highest concentration of galectin-9 increased MCP-1 levels in the blood plasma. However, these concentrations of MCP-1 are not elevated enough to induce basophil degranulation, since they are approximately 0.02–0.05 nM. In addition, no correlation was observed between MCP-1 levels and basophil degranulation, and no spontaneous CD63 release was observed after 2, 6, and 24 h of pre-incubation with NDOs or galectin-9 (data not shown). This might indicate that NDOs and galectin-9 can have direct effects on basophils. A previous study investigating the effects of galectin-9 on degranulation of HMC-1, a mast cell line that does not express FcεRI, also indicated an increase of MCP-1, which was dose-dependently correlated to increased galectin-9 levels (42). In this study, galectin-9 reduced PMA and ionomycin-induced degranulation of HMC-1 and induced the phosphorylation of the ERK1/2 pathway. Based on these results, it would be interesting to investigate whether activation of this ERK1/2 signaling pathway is also involved in the reduction in degranulation like we observed in human basophils.

This study was an *in vitro* model, and it is therefore important that the effects observed in this study are validated *in vivo* in humans. For future research, it would be interesting to focus on the effects of NDOs and other components on other cells subsets, such as eosinophils, that play an important role in allergy and also have degranulation capacities. In addition, this study was performed in whole blood and results shown may be caused by either a direct or indirect effect of NDOs. To be able to investigate the mechanism of action of these NDOs and galectin-9 on basophil degranulation, and to disseminate between these paths, it would be interesting to perform these experiments on isolated basophils of allergic patients, to exclude the involvement of surrounding cells.

In conclusion, this study indicated that NDOs can decrease basophil degranulation in an *in vitro* model. Indirect mediator galectin-9, which may be released by different kinds of cell types in response to exposure to NDOs, was also able to decrease basophil degranulation. No modification of the FcεRI receptor expression, cytokines, or chemokines was found in relation to this effect. The exact mechanism of action by which these NDOs can exert their immunomodulatory effects still needs to be elucidated, and *in vivo* validation of these results is necessary. However, it indicates that these NDOs are interesting dietary immunomodulatory agents, and might be useful as adjunct therapy in allergen-specific immunotherapy.

## Ethics Statement

All patients gave written informed consent before enrollment in the study. The study was reviewed and approved by the Ethics Committee of the University Medical Center Utrecht (NL51606.041.15).

## Author Contributions

SH, LW, and HO designed the experiments; AK assisted in recruitment of peanut-allergic patients; SH and CJ performed the experimental procedures. SH interpreted and collected data and drafted the manuscript. EK, JG, AK, LW, and HO contributed to data interpretation and critically revised the manuscript.

## Conflict of Interest Statement

JG is partly employed by Danone Nutricia Research. SH, CJ, AK, EK, LW, and HO: no conflict of interest.
